# Population pharmacokinetics of meropenem in critically ill patients

**DOI:** 10.1515/med-2024-1004

**Published:** 2024-07-22

**Authors:** Aleksandar Rančić, Miloš N. Milosavljević, Nikola Rosić, Dragan Milovanović, Marko Folić, Dejana Ružić Zečević, Nemanja Petrović, Mirjana Milojević Čorbić, Vera Dabanović, Slobodan M. Janković

**Affiliations:** Institute of Public Health Kragujevac, Kragujevac, Serbia; Department of Pharmacology and Toxicology, Faculty of Medical Sciences, University of Kragujevac, Svetozara Markovica 69, Kragujevac, 34000, Serbia; Clinical Pharmacology Department, University Clinical Centre Kragujevac, Kragujevac, Serbia; Department of Pharmacy, Faculty of Medical Sciences, University of Kragujevac, Kragujevac, 34000, Serbia; Pharmacy Institution of Montenegro “Montefarm”, Podgorica, Montenegro

**Keywords:** population pharmacokinetics, meropenem, critically ill patients, NONMEM software

## Abstract

**Objective:**

The pharmacokinetics of meropenem are significantly altered in critically ill patients. A population pharmacokinetic study was designed to estimate typical values of meropenem clearance in critically ill patients and evaluate potential factors of influence.

**Methods:**

After meropenem reached a steady state in each patient, two blood samples were taken within the dose interval. The one-compartment pharmacokinetic model based on the data from 101 intensive care unit patients was built using NONMEM software.

**Results:**

Typical values of meropenem clearance and volume of distribution were 3.80 L/h and 3.52 L, respectively. In the final model, meropenem clearance was influenced by serum concentrations of creatinine (CRE), leukocyte count (WBC), hypertension (HTA), and concomitant use of vancomycin (VAN) or colistimethate (COL): CL (L/h) = 5.29 × CRE ^ 0.000001 × WBCs ^ (−0.165) + 0.000001 × HTA + 0.825 × VAN + 1.28 × COL.

**Conclusion:**

In order to achieve effective plasma concentrations of meropenem in critically ill patients, the meropenem dosing regimen should be adjusted according to individual values of drug clearance.

## 1 Introduction

Meropenem is an antibiotic from the group of carbapenems that achieves its bactericidal effect by inhibiting the synthesis of the cell wall of bacteria [1] and by inhibiting penicillin-binding proteins (PBPs); it binds covalently to PBP2, 3, and 4, disabling the synthesis of the bacterial wall [2]. Meropenem has the highest affinity for PBP2 and PBP4, and its concentration causes 50% inhibition (IC_50_) below 0.0075 mg/L in Gram-negative bacteria [3]. It is particularly effective against Gram-negative bacteria but also has a significant effect on most Gram-positive bacteria and some strains of anaerobic bacteria [4]. Meropenem is primarily used for the treatment of serious hospital-acquired infections, like pneumonia, intra- and postpartum infections, acute bacterial meningitis, complicated intra-abdominal, urinary, skin, and soft tissue infections, and sepsis [5,6]. It significantly decreases the mortality of critically ill patients with sepsis, especially if administered as a continuous infusion (19 vs 37%) [7]. Meropenem is administered intravenously, rapidly penetrates most bodily tissues, and is eliminated mostly in urine as an unchanged drug [6,8]. The volume of distribution of meropenem is 21 L [8]. In patients with normal renal function, the elimination half-life of meropenem is approximately 1 h [6].

Meropenem is frequently prescribed to critically ill patients due to its efficiency and broad spectrum of activity [9]. However, it is known that the pharmacokinetics of meropenem is significantly altered in critically ill patients [10], which could result in reduced antimicrobial efficacy or increased drug toxicity. Several population pharmacokinetic (PPK) studies have been conducted to date, and it has been determined that some factors have a substantial impact on the clearance and volume of distribution of meropenem in critically ill patients [11,12,13]. Meropenem clearance in critically ill patients has been shown to be influenced by gender [11], age [12], creatinine clearance [12,13], and sepsis [11]; while factors such as serum albumin concentration [11,13], shock [13], age [11], and body weight [12] affect the volume of distribution of meropenem in the critically ill patients. However, the effects of concomitant antibiotics on the pharmacokinetics of meropenem in critically ill patients have not yet been thoroughly investigated. However, it could be of great clinical significance since critically ill patients often receive a combination of antibiotics [14].

Therefore, we conducted a PPK study to examine the influence of several potential factors that may affect the clearance of meropenem in critically ill patients.

## 2 Materials and methods

### 2.1 Design and study population

This was a prospective observational study of the case-series type. The study was carried out on critically ill patients who received meropenem by intermittent intravenous infusion during treatment in the Intensive Care Unit (ICU) at the University Clinical Center Kragujevac (UCCKG), Serbia. Only critically ill patients with severe infections (meningitis, pneumonia, sepsis, septic shock, and febrile neutropenia) caused by multi-resistant Gram-negative bacteria and treated in ICUs were included in the study. The diagnosis of the infection was made by infectious disease specialists on the basis of clinical and laboratory parameters. Meropenem was administered in doses of 1,000–2,000 mg every 8 or 12 h. The Ethics Committee of UCCKG had approved the study before its initiation (No. 01-21-63).

The study included only those critically ill patients in whom a steady state of meropenem was achieved, which implied continuous administration of meropenem for at least 3 days. The exclusion criteria were as follows: absence of critical illness; duration of meropenem therapy less than 3 days; pregnant and lactating women; and refusal of a patient or his/her legal representative to sign the informed consent form. The study sample was convenient but consecutive, i.e., all patients of the target population who met the inclusion criteria during the study period but did not meet the exclusion criteria were included.

We collected the following demographic and clinical data for each patient included in the study: total body weight (TBW), age, gender, total daily dose of meropenem, a dosing schedule of meropenem, time of the last meropenem intake, values of basic biochemical parameters (creatinine, aspartate aminotransferase, alanine transaminase, red blood cell count, hemoglobin, white blood cell count, platelet count), the presence of comorbidities (hypertension, chronic renal failure, malignant diseases, cerebral infarction, pneumonia, urinary tract infections), and data about antibiotics and other drugs that patients received at the same time as meropenem.

### 2.2 Drug analysis

After meropenem reached a steady state, two 5 mL blood samples were taken from each patient by puncture of the cubital vein. The first sample was taken 5–30 min after the end of the intravenous infusion of meropenem, while the second sample was taken 3–4 h after the end of drug infusion. The samples were centrifuged at 3,000 rpm to separate the plasma, which was frozen and stored at −20°C until the meropenem concentration was measured.

Meropenem plasma concentrations were measured using the high-performance liquid chromatography (HPLC) method in the analytical laboratory within the Institute of Public Health in Kragujevac. We used an HPLC system from Agilent Technologies 1200 Series (Agilent Technologies, USA) equipped with a binary pump (G1312A), an online solvent degasser (G1379B), and an autosampler (G1329A), a thermostatically controlled column compartment (G1316A), coupled with a diode array detector (G1315D). Chromatographic data collection and processing were performed using HP ChemStation software. Separation was performed at 25°C using a Zorbax Eclipse XDB-C18 column (150 mm × 4.6 mm, 5 μm) by Agilent Technologies (USA), and protected by Security Guard C18 cartridge (12.5 mm × 4.6 mm, 5 μm), which is installed pre-column. The mobile phase was composed of 0.1 M phosphate buffer (pH 7) and methanol (80:20, v/v) in an isocratic elution mode. The flow rate of the mobile phase was 1.0 mL/min, and the detection wavelength was 300 nm. Methanol (HPLC grade) was obtained from J.T. Baker (Deventer, Netherlands), while di-sodium hydrogen phosphate dihydrate (Na_2_HPO_4_·2H_2_O), potassium dihydrogen phosphate (KH_2_PO_4_), and orthophosphoric acid (H_3_PO_4_, 85%) were purchased from Merck (Darmstadt, Germany). The validation procedure of our HPLC method for the measurement of meropenem was performed in accordance with official American and European guidelines for bioanalytical method validation through the assessment of the linearity and limit of detection/quantification, accuracy and precision, recovery, stability, specificity, and selectivity [15,16].

### 2.3 Pharmacokinetic analysis

A PPK model for the clearance of meropenem was developed using NONMEM software with the selection of the ADVAN 1 subroutine (version 5, level 1.1, double precision) [17]. It implies the application of a single-compartment model without absorption.

The development of a PPK model for the clearance of meropenem is a complex process consisting of three phases. The first step of this analysis was to obtain a basic structural model (without examining the influence of covariates) for the clearance of meropenem using the appropriate subroutine from the PREDPP library of NONMEM. The software builds a model that predicts plasma concentrations of meropenem, and then the predicted values are compared with measured values; the differences are further minimized in an iterative process through adjustment of the model. In this way, by analyzing the entered data, a typical mean value of meropenem clearance in the examined population was obtained.

In the second phase, the full model was built through individual inclusion of covariates (TBW, age, gender, total daily dose of meropenem, values of basic biochemical parameters, comorbidities, and co-medication with other antibiotics) in the base model, one by one. Both linear and non-linear methods of examining the effect of covariates on meropenem clearance were used. As a result, a large number of univariate models were obtained. To assess whether univariate models significantly improved data fitting, the value of the minimum objective function (MOF), which represents the negative double logarithm of the probability of the data (2 loglikelihood, 2 LL), was used. If a covariate’s impact on the base model was followed by a reduction in the MOF value of at least 3.84 (*p* < 0.05), it was considered significant [18]. After that, the process of simultaneous inclusion of all statistically significant covariates followed, which led to the formation of a full model.

To obtain the final model for meropenem clearance, a backward deletion process was performed for each of the covariates from the full model. In this process, it was necessary to satisfy much stricter statistical criteria (increase in MOF greater than 6.64 for *p* < 0.01) to obtain the covariates of the final model. The final pharmacokinetic model of meropenem clearance consisted of only the factors that fulfilled this requirement.

The validity of the final pharmacokinetic model of meropenem clearance in the studied population was evaluated by the following: reduction in inter- and intra-individual (residual) variability, improvement of scatter plots of predicted values (PRED) versus observed concentrations (DV), and weighted residuals (WRES) versus predicted concentrations (PRED), from the base to the final model. Exponential and additive error models were used to evaluate the estimation of intra-individual variability in clearance and residual error in concentration.

At the end of the PPK analysis, the final model was additionally validated by bootstrapping to evaluate its predictive performance and the possibility of clinical application. This non-parametric method uses a resampling methodology that involves a sizable number of data replications (a few hundred or thousands), replacement from the index set, and sampling from individual patients as the sampling unit. Each bootstrap data set was fitted to the final model to produce the bootstrap estimated values of the pharmacokinetic parameters, and NONMEM software was used to examine the variability of these values. The calculated mean values of the estimated pharmacokinetic parameters, together with the 2.5–97.5th percentiles from the bootstrap dataset, were compared with the estimates of the pharmacokinetic parameters from the final model.

### 2.4 Statistical analysis

Frequency and percentages were used to describe categorical variables. Continuous variables were represented by the mean, standard deviation, and range. The calculated and presented estimates of the model coefficients were reported as means with 95% confidence intervals (±1.96 × the standard error of the estimate). For the estimated values of pharmacokinetic parameters from the bootstrap dataset, 2.5 and 97.5 percentiles were calculated. The SPSS software package, version 22, was used for all of those calculations.

## 3 Results

A total of 101 critically ill patients were included in this study. The baseline demographic and clinical characteristics of our patients are shown in Table 1.

**Table 1:** Demographic and clinical data for patients included in population analysis

Parameter	Mean values ± SD	Range
Number of patients	101	
Number of observations	202	
Gender (male/female)	62/39 (61.4%/38.6%)	
TBW (kg)	78.97 ± 13.76	48–130
Age (years)	62.37 ± 14.89	21–86
CRE (µmol/L)	94.47 ± 63.68	40–452
MER dose (mg/day)	3,000 ± 692.82	2,000–6,000
MER plasma concentration (*C*_1_) (µg/mL)	40.69 ± 16.67	13.07–88.95
MER plasma concentration (*C*_2_) (µg/mL)	12.55 ± 7.62	2.06–36.28
MER monotherapy	74 (73.3%)	
MER polytherapy with	27/(26.7%)	
Vancomycin	17/(16.8%)	
Colistin	7/(6.9%)	
Vancomycin and colistin	3/(2.97%)	
Comorbidities of patients with		
HTA	33/(32.7%)	
CRF	12/(11.9%)	
NEO	18/(17.8%)	
CI	21/(20.8%)	
PNE	32/(31.7%)	
UTI	36/(35.6%)	

TBW – total body weight; CRE, creatinine concentration (µmol/L); MER – meropenem; *C*_1_ – concentration of meropenem 5 min after end of intravenous infusion; *C*_2_ – concentration of meropenem 3 h after intravenous infusion; HTA, hypertension; CRF, chronic renal failure; NEO, neoplasm; CI, cerebral infarction; PNE, pneumonia; UTI, urinary tract infection.

The estimated values for clearance and volume of distribution of meropenem in the base model were 3.80 L/h and 3.52 L, respectively. The value of MOF in the base model was 1283.053.

In the next step, we examined the influence of individual covariates on the clearance of meropenem. It was found that 10 out of the 24 examined covariates had a statistically significant influence on the MOF value in the base model. Therefore, the full PPK model of meropenem had 13 significant covariates: age, TBW, daily dose of meropenem, creatinine, white blood cells, presence of hypertension, presence of chronic renal failure, presence of neoplasm, presence of cerebral infarction, presence of pneumonia, presence of urinary tract infection, concomitant administration of vancomycin, and concomitant administration of colistimethate (Table 2). All other covariates were excluded in this process. The MOF value in the full model after including all covariates with a significant impact was 1207.153.

**Table 2:** MOF values in the base model, univariate model, and full model

Clearance models	MOF	Difference in MOF	*p* value^⁎⁎^
Base model			
CL = *θ*_1_ × Exp[ETA(1)]	1283.053		
Univariate models			
CL = *θ*_1_ × (AGE/50) ^ *θ*_3_ × Exp[ETA(1)]	1277.958	5.095	<0.05
CL = *θ*_1_ × (TWB/70) ^ *θ*_4_ × Exp[ETA(1)]	1266.640	16.413	<0.01
CL = *θ*_1_ × (DD) ^ *θ*_5_ × Exp[ETA(1)]	1282.624	0.429	>0.05
CL = *θ*_1_ × (CRE) ^ *θ*_6_ × Exp[ETA(1)]	1272.552	10.501	<0.01
CL = *θ*_1_ × (WBCs) ^ *θ*_7_ × Exp[ETA(1)]	1267.150	15.903	<0.01
CL = (*θ*_1_ + *θ*_8_ × HTA) × Exp[ETA(1)]	1262.636	20.417	<0.01
CL = (*θ*_1_ + *θ*_9_ × CRF) × Exp[ETA(1)]	1282.954	0.099	>0.05
CL = (*θ*_1_ + *θ*_10_ × NEO) × Exp[ETA(1)]	1280.925	2.128	>0.05
CL = (*θ*_1_ + *θ*_11_ × CI) × Exp[ETA(1)]	1264.258	18.795	<0.01
CL = (*θ*_1_ + *θ*_12_ × PNE) × Exp[ETA(1)]	1275.855	7.198	<0.01
CL = (*θ*_1_ + *θ*_13_ × UTI) × Exp[ETA(1)]	1272.183	10.870	<0.01
CL = (*θ*_1_ + *θ*_14_ × VAN) × Exp[ETA(1)]	1272.001	11.052	<0.01
CL = (*θ*_1_ + *θ*_15_ × COL) × Exp[ETA(1)]	1274.813	8.240	<0.01
Full model			
CL = [*θ*_1_ × (AGE/50) ^ *θ*_3_ × (TWB/70) ^ *θ*_4_ × (CRE) ^ *θ*_6_ × (WBCs) ^ *θ*_7_ + *θ*_8_ × HTA + *θ*_11_ × CI + *θ*_12_ × PNE + *θ*_13_ × UTI + *θ*_14_ × VAN + *θ*_15_ × COL] × Exp[ETA(1)]	1207.153		

CL – clearance (L/h); *θ*1 – typical value of CL; ETA(1) – inter-individual variability in CL; *θ*_3_ to *θ*_15_ – slopes of the covariate effects; AGE – patient age (years); TBW – total body weight (kg); DD – total daily dose of meropenem (mg/day); CRE – creatinine concentration (µmol/L); WBCs – white blood cells; HTA – hypertension; CRF – chronic renal failure; NEO – neoplasm; CI – cerebral infarction; pneumonia; PNE – UTI – urinary tract infection; VAN – vancomycin; COL – colistin; MOF – minimum objective function; ^*⁎⁎*^ *p-*values for the MOF difference between the base and tested models.

Using the backward deletion technique with stricter statistical parameters of significance, the extent of the influence of the covariates that were significant in the univariate models was again evaluated. It was found that 5 out of 10 individual covariates from the full model met the necessary statistical requirements: creatinine, white blood cell count, presence of hypertension, concomitant use of vancomycin, and concomitant use of colistimethate. Therefore, these five covariates were entered into our final model for meropenem clearance:

\[\text{CL}(\text{L}/\text{h})=5.29\times {\text{CRE}}^{\wedge }0.000001\times {\text{WBCs}}^{\wedge }(-0.165)+0.000001\times \text{HTA}+0.825\text{}\times \text{VAN}+1.28\text{}\times \text{COL,}]\]

where CL represents clearance, CRE represents creatinine, WBC represents leukocyte count, HTA represents hypertension, VAN represents concomitant use of vancomycin, and COL represents colistimethate.

The values of the estimated parameters of the final model are shown in Table 3. The MOF value of our final model is statistically significantly lower than the MOF value in the base model (1224.035 vs 1283.053, *p* < 0.01). Inter- and intra-individual variability were 2.15 and 44%, respectively.

**Table 3:** Parameter estimates for the final model

Model parameter	Estimated value	Standard error	95% confidence interval
Clearance (L/h) (*θ*_1_)	5.29	0.038	5.215–5.365
Volume of distribution (L) (*θ*_2_)	2.05	0.006	2.039–2.061
Effect of CRE (× (CRE) ^ *θ*_6_)	0.0000	0.0000	−0.0000–0.0000
Effect of WBCs (× (WBCs) ^ *θ*_7_)	−0.165	0.05450	−0.219–(−0.110)
Effect of HTA (+ *θ*_8_ × HTA)	0.0000	0.0000	−0.0000–0.0000
Effect of VAN (+ *θ*_14_ × VAN)	0.825	0.054	0.770–0.879
Effect of COL (+ *θ*_15_ × COL)	1.28	0.05	1.23–1.33
Inter-individual variance of CL (*ω* ^2^_CL_)	0.0215	0.0146	0.0000–0.0501
Residual error variance (*σ* ^2^_CL_)	0.44	0.066	0.310–0.570

**Figure 1:**
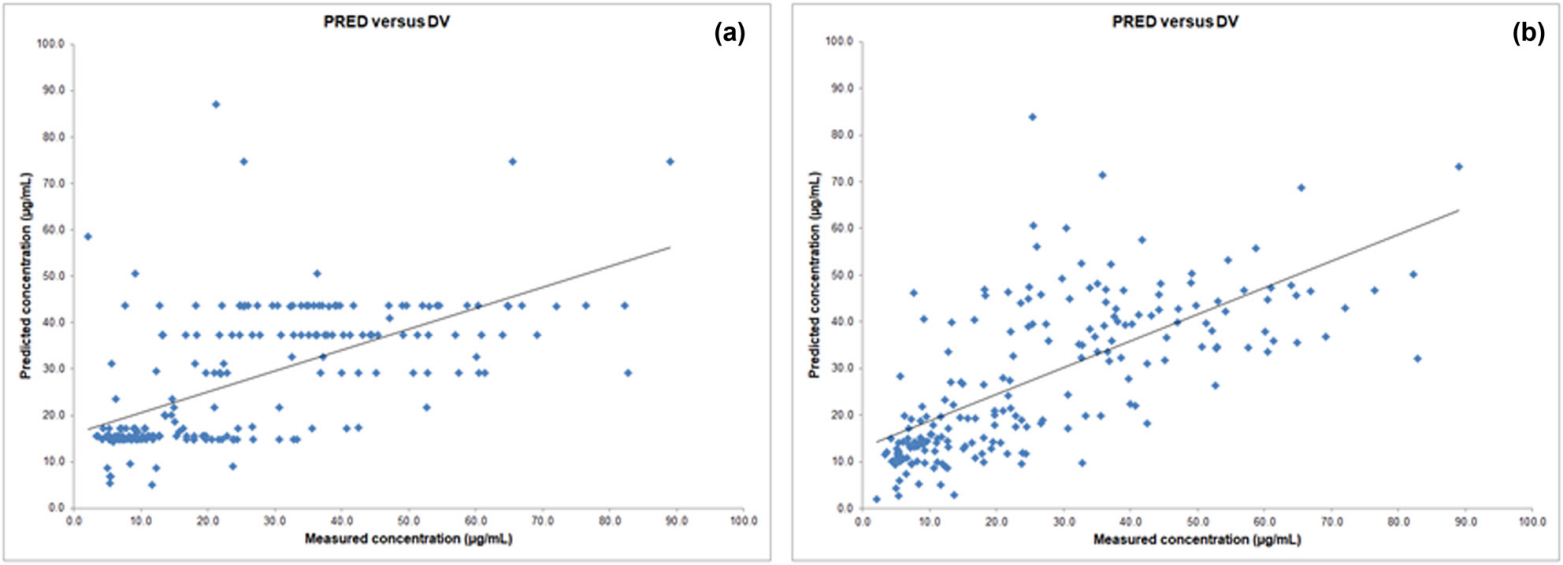
Scatter plot of predicted concentrations (PRED) versus measured concentrations (DV) for meropenem by the base model (a) and the final model (b) in the target population. Concentrations of meropenem are expressed in µg/mL.

**Figure 2:**
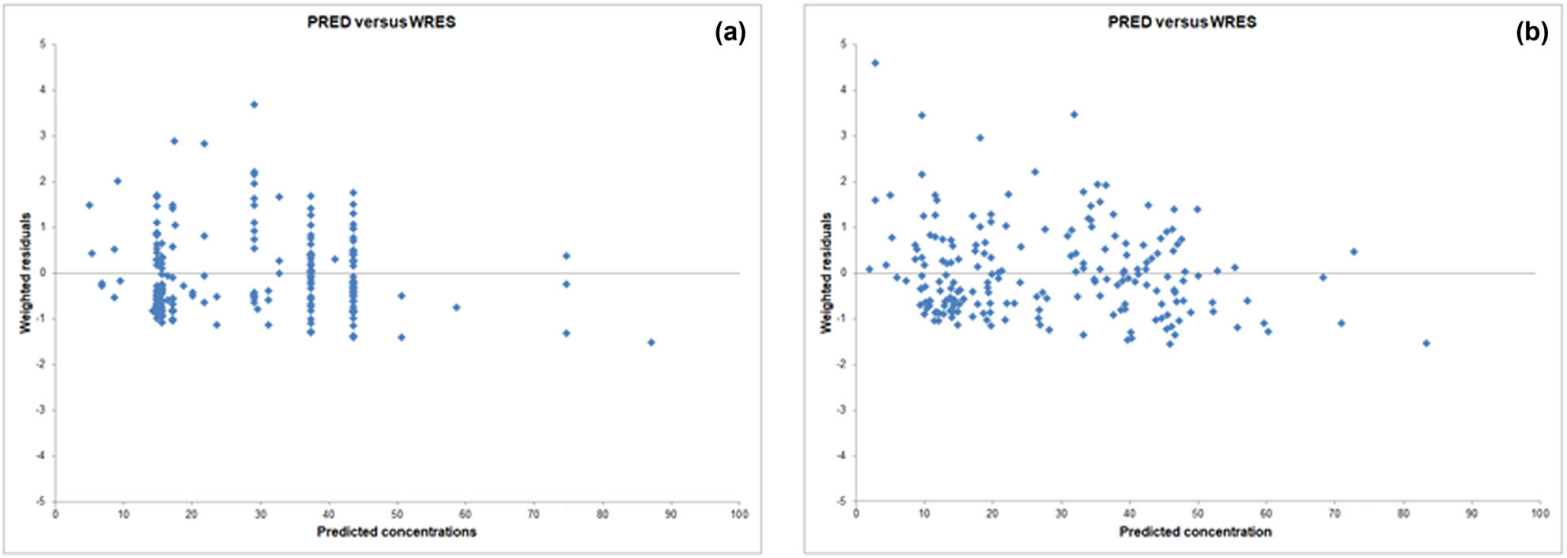
Scatter plot of weighted residuals versus predicted concentrations (PRED) for meropenem by the base model (a) and the final model (b) in the target population.

The improved distribution of data in the final model compared to the base model is shown in Figure 1, which shows the relationship between predicted (PRED) and measured serum concentrations (DV) of meropenem. Figure 2 shows the ratio of WRES versus predicted concentrations (PRED) for meropenem by the base model and the final model. It can be seen that the distribution of individual points around zero is much improved in the final model compared to the base model.

To assess the accuracy of the models, we used the mean squared prediction error (MSPE) and the root mean squared prediction error (RMSPE), while for the assessment of the bias, we calculated the mean prediction error (MPE). The calculated prediction errors for the base, full, and final models are shown in Table 4.

**Table 4:** Prediction errors ± 95% confidence interval

Error	Base model	Full model	Final model
MPE	11.60 (8.55–14.65)	10.84 (7.95–13.73)	10.91 (7.80–14.02)
MSPE	242.83 (141.37–344.30)	217.94 (135.15–300.73)	253.06 (94.67–411.45)
RMSPE	15.56	14.75	15.86

A bootstrapping analysis with 100 bootstrap runs was carried out as part of the validation process. The results of the bootstrapping analysis indicate the reliability and stability of our model since the mean estimated values of clearance and volume of distribution of meropenem calculated by bootstrapping are comparable to the mean estimated values of these pharmacokinetic parameters from the original NONMEM analysis (Table 5).

**Table 5:** Bootstrapping analysis of base and final models

Model parameter	Mean values ± SD	Standard error	95% confidence interval
Base model			
Clearance (L/h) (*θ*_1_)	4.091 ± 0.159	0.016	4.060–4.122
Volume of distribution (L) (*θ*_2_)	3.239 ± 0.136	0.014	3.212–3.266
Final model			
Clearance (L/h) (*θ*_1_)	3.864 ± 0.186	0.019	3.827–3.900
Volume of distribution (L) (*θ*_2_)	1.809 ± 0.565	0.057	1.699–1.920
Effects on clearance			
Effect of CRE (×(CRE) ^ *θ*_6_)	0.000 ± 0.000	0.000	0.000–0.000
Effect of WBCs (×(WBCs) ^ *θ*_7_)	0.001 ± 0.001	0.000	0.000–0.002
Effect of HTA (+*θ*_8_ × HTA)	0.059 ± 0.166	0.017	0.026–0.091
Effect of VAN (+*θ*_14_ × VAN)	0.660 ± 0.560	0.056	0.550–0.769
Effect of COL (+*θ*_15_ × COL)	0.662 ± 0.615	0.061	0.541–0.782
Effects on the volume of distribution			
Effect of CRE (×(CRE) ^ *θ*_16_)	0.130 ± 0.199	0.020	0.091–0.169
Effect of WBCs (×(WBCs) ^ *θ*_17_)	0.021 ± 0.034	0.003	0.015–0.028
Effect of HTA (+*θ*_18_ × HTA)	0.170 ± 0.205	0.021	0.130–0.210
Effect of VAN (+*θ*_19_ × VAN)	0.577 ± 0.524	0.052	0.474–0.680
Effect of COL (+*θ*_20_ × COL)	0.581 ± 0.604	0.060	0.462–0.699

CL – clearance (L/h); *θ*_1_ – the typical value of CL; ETA(1) – inter-individual variability in CL; *V* – the volume of distribution (L); *θ*_2_ – the typical value of *V*; ETA(2) – inter-individual variability in *V*; *θ*_6_, *θ*_7_, *θ*_8_, *θ*_14_, and *θ*_15_ – slopes of covariate effects to estimate the clearance of meropenem; *θ*_16_–*θ*_20_ – slopes of covariate effects to estimate the volume of distribution of meropenem; CRE – creatinine concentration (µmol/L); WBCs – white blood cells; HTA – hypertension; VAN – vancomycin; COL – colistin.

## 4 Discussion

We developed a single-compartment PPK model for meropenem clearance in a critically ill patient population that showed satisfactory accuracy and stability. The factors that are significantly associated with increased clearance of meropenem in this patient population were the serum concentration of creatinine, the number of white blood cells, the presence of hypertension, the concomitant use of vancomycin, and the concomitant use of colistimethate.

It is very difficult to estimate renal clearance of drugs in critically ill patients. This is primarily because, in a significant number of critically ill patients, the phenomenon of augmented renal clearance occurs [19,20,21]. Augmented renal clearance indicates a condition that is accompanied by creatinine elimination rates higher than 130 mL/min/1.73 m^2^ and increased clearance of medications eliminated via kidneys [22,23]. This phenomenon of enhanced renal elimination can be found in critically ill patients suffering from various diseases, especially those with severe infections, sepsis, burns, trauma, subarachnoid hemorrhage, and hematological malignancy [20,21]. Several potential mechanisms may contribute to the occurrence of augmented renal clearance, including the influence of inflammatory mediators released during the systemic inflammatory response syndrome, alterations in neurohormonal balance, and increased use of fluids and vasoactive drugs [19,21]. In addition to an increased glomerular filtration rate, augmented renal clearance is also accompanied by enhanced tubular secretion [24].

In this study, higher serum concentrations of creatinine were associated with larger meropenem clearance. This finding could seem controversial at first, especially in light of the widespread belief that elevated serum creatinine levels indicate impaired kidney function [25]. Although serum creatinine is an essential factor in assessing renal function, there are important practical limitations to its use [26], so it is not the best predictor of renal function [27]. Besides, the average serum creatinine values also showed that the vast majority of the patients in our study did not have any kind of renal impairment. In any case, the effect of serum creatinine on meropenem clearance that we observed in our study does not seem to have major clinical implications since previous studies have shown that, despite normal serum creatinine concentrations, a significant percentage of critically ill patients show enhanced renal clearance [28,29,30].

It is well known that patients with severe infections or sepsis have a greater clearance of antibiotics that are predominantly eliminated by the renal route [31]. This is attributed to the phenomenon of augmented renal clearance [32]. It is believed that the increased renal clearance of drugs in conditions of sepsis or severe infections is due to the increased production of cytokines and other pro-inflammatory mediators that reduce vascular resistance, increase cardiac response, and increase blood flow through the kidneys [32,33,34]. However, white blood cells make up a significant compartment in the human organism where drugs may enter and be retained in various quantities, influencing their overall pharmacokinetics [35]. During sepsis, white blood cells are activated with increased metabolism, which may decrease their capacity to retain drugs. Therefore, it is not surprising that our results indicate that plasma levels of white blood cells, which are elevated in sepsis and severe infections [36], are associated with greater clearance of meropenem.

The results of this study also revealed a statistically significant positive relationship between hypertension and the clearance of meropenem. It is well known that high blood pressure increases renal vascular resistance and decreases renal blood flow [37]. However, the glomerular filtration rate remains normal, while the filtration fraction even increases in conditions of hypertension [37]. Fluid replacement therapy and vasoactive medications are often administered to critically ill patients as a part of usual management to increase blood pressure [38]. Given that fluid replacement therapy and vasoactive medications are mentioned as potential causes of the occurrence of augmented renal clearance in critically ill patients [19,21], it is most likely that hypertension does not have a specific effect on the clearance of meropenem but rather that this effect is manifested as part of the augmented renal clearance.

Given that combinations of antibiotics are often used in critically ill patients [14], the results of this study, which indicate that the clearance of meropenem is higher in the case of simultaneous administration of vancomycin or colistimethate, are of particular clinical importance. There are numerous ways in which concomitant medication may influence the pharmacokinetics of an investigated drug: through induction or inhibition of drug-metabolizing enzymes, through induction or inhibition of drug transporters in cell membranes, by interactions with plasma proteins, etc. However, the influence on the pharmacokinetics of concomitant drugs may also be only apparent since it may coincide with other pathophysiological factors that truly influence pharmacokinetics, like augmented renal clearance. Patients who require a combination of reserve antibiotics also have a more severe infection, which is always accompanied by augmented renal clearance in the beginning. Augmented renal clearance is a condition that can lead to subtherapeutic concentrations of renally excreted antibiotics [21], such as meropenem, vancomycin, and colistimethate. Particularly interesting is the case of a critically ill patient in whom meropenem and vancomycin were used for the treatment of periodontitis, endophthalmitis, and multiple brain abscesses caused by *Streptococcus intermedius* [39]. Due to enhanced elimination, adequate plasma concentrations and an antimicrobial effect were achieved only at doses of meropenem and vancomycin of 16 and 6 g/day, respectively [39]. The fact that serum creatinine levels and glomerular filtration rate were within normal ranges suggested that enhanced tubular secretion was the cause of the higher renal clearance of antibiotics in this patient [39]. Meropenem undergoes both glomerular filtration and tubular secretion in the kidneys [6]. Therefore, an increase in either of these two processes may result in meropenem’s augmented elimination.

To the best of our knowledge, we are the first to have observed that the simultaneous administration of meropenem with vancomycin or colistimethate enhances the clearance of this carbapenem antibiotic in critically ill patients. However, a relatively small number of our patients received meropenem along with vancomycin and colistimethate, which is the main limitation of our study. Additional limitations of the study are the small number of measurements of meropenem plasma concentrations per patient and the limited number of variables that were investigated in the model. These limitations were caused by the acute nature of infections treated by meropenem in critically ill patients, which precluded the collection of some more elaborate laboratory values or additional diagnostics. All mentioned limitations decreased the statistical power of our study, creating the possibility that we missed some factors that could have affected the pharmacokinetics of meropenem significantly. There are several directions in which future research should probably go. First, larger studies with more patients and more comprehensive data collection could reveal additional factors that influence the clearance of meropenem and patients with bacterial infections, which could have been missed in this study. Second, certain subpopulations of patients should be investigated separately, especially those with renal impairment and liver failure, since they have a much different interior milieu for the movement and action of meropenem, making it probably more sensitive to the influences of other drugs or comorbidities. The population of obese and morbidly obese critically ill patients may also be of interest since Alobaid et al. showed in their study of the PPK of meropenem that body mass index was a significant covariate describing meropenem volume of distribution in the central compartment [40]. Third, additional studies are also needed to investigate the mechanism by which vancomycin and colistimethate increase the clearance of meropenem in critically ill patients. The results of future studies would ultimately help clinicians make the basis for more precise dosing of meropenem in critically ill patients.

## 5 Conclusion

The clearance of meropenem in critically ill patients was positively correlated with the serum creatinine level, the white blood cell count, the presence of hypertension, the concomitant use of vancomycin, and the concomitant use of colistimethate. Therefore, in order to achieve effective plasma concentrations of meropenem in critically ill patients, the meropenem dosing regimen should be adjusted according to individual values of drug clearance. A significant portion of critically ill patients may experience the phenomenon of augmented renal clearance, which, in the presence of known factors that increase the clearance of meropenem, may result in the achievement of subtherapeutic concentrations of this antibiotic and the consequent lack of therapeutic effect and increased mortality in these patients, unless the dosing regimen is adjusted accordingly
